# Effects of Dietary Isoflavones from *Puerariae* Radix on Lipid and Bone Metabolism in Ovariectomized Rats

**DOI:** 10.3390/nu5072734

**Published:** 2013-07-17

**Authors:** Dong Wook Lim, Jae Goo Kim, Yun Tai Kim

**Affiliations:** Functionality Evaluation Research Group, Korea Food Research Institute, Seongnam 463-746, Korea; E-Mails: neodw4015@naver.com (D.W.L.); yadj2654@naver.com (J.G.K.)

**Keywords:** *Pueraria lobata*, isoflavone, lipid metabolism, bone, ovariectomized

## Abstract

*Puerariae* radix, the dried root of *Pueraria lobata* Ohwi, is one of the earliest and most important edible crude herbs used for various medical purposes in oriental medicine. This study evaluated the metabolic effects of total isoflavones from *P. lobata* (PTIF) in ovariectomized (OVX) rats. The OVX rats were divided into four groups treated with distilled water, 17β-estradiol (E2 10 μg/kg, once daily, i.p.) and PTIF (30 and 100 mg/kg, once daily, p.o.) for eight weeks. The treatments with high-dose PTIF significantly decreased the bone mineral density (BMD) loss in the femur and inhibited the increase in body weight and lipoprotein levels compared to the OVX-control group without elevating the serum levels of the liver enzymes, aspartate aminotransferase (AST) and alanine transaminase (ALT). Furthermore, PTIF exhibits a hepatoprotective effect in OVX-induced hepatic steatosis, indicated with reduced hepatic lipid contents. Taken together, our findings suggest that PTIF may be useful for controlling lipid and bone metabolism, at least in OVX rats. Further research is necessary to determine whether PTIF will have the same effects in humans.

## 1. Introduction

Menopause is often associated with serious public problems in middle-aged women; thus, appropriate care and management of health after menopause is important to maintaining a woman’s quality of life [1]. A reduction in estrogen levels is commonly believed to cause psychological and mood changes, as well as physiological changes that result in symptoms, such as osteoporosis, breast cancer, hot flashes, obesity, hyperlipidemia and cardiovascular disease [2]. These menopausal symptoms can be at least comparatively reversed by the local or systemic administration of exogenous estrogens; thus, hormone replacement therapy (HRT) has been used to ameliorate menopausal symptoms [3]. However, long-term HRT increases the risk of several serious diseases, such as breast and endometrial cancer, thromboembolic events and vaginal bleeding [4,5]. Therefore, alternative approaches, including dietary interventions, have been of interest in the control of menopausal symptoms.

Phytoestrogens are naturally occurring hormone-like compounds found in plants and can be subdivided into coumestans, isoflavonoids and lignans. Coumestrol and the isoflavonoids genistein, daidzein, and their plant precursors, are mainly found in soybeans and clover [6]. Isoflavones, especially those derived from plants, have various biological activities and can improve the metabolic symptoms [7] and bone-protective effects [8] of menopause. Although some studies have suggested that isoflavones have potential health benefits in postmenopausal women [9], a number of studies have also reported the absence of beneficial effects of isoflavones intake on classic metabolic parameters [10], such as body weight and serum lipid profiles. A number of randomized trials have also failed to show a sustained enhancement of bone by isoflavones supplements [11,12,13]. Since we currently lack consistent evidence to support any beneficial effect of isoflavones intake on menopausal symptoms, their efficacy needs to be scientifically evaluated in *in vivo* experiments.

*Puerariae* radix, the dried root of *Pueraria lobata* Ohwi, is one of the earliest and most important edible crude herbs used for various medical purposes in oriental medicine. *Puerariae* radix has been reported to have antioxidant [14], hypoglycemic [15] and anti-thrombosis effects [16], as well as lowering plasma cholesterol [17]. The major active components of *Puerariae* radix are flavonoids, coumarins and isoflavones, such as daidzein, genistein and puerarin, and they are responsible for its diverse pharmacological activities [18].

In the present study, we examined the effects of the *P. lobata* total isoflavones (PTIF), which contain the unique isoflavone, puerarin, on menopausal symptoms. Puerarin has shown relatively strong estrogenic activities compared with other isoflavones; thus, we hypothesized that puerarin-containing isoflavones may have a significant protective effect against menopausal symptoms, which often include dyslipidemia and osteoporosis. We used ovariectomized (OVX) rat, which exhibit most of the characteristics of human menopausal symptoms [19], treated with PTIF during an eight-week experimental period and then assessed the metabolic parameters related to menopausal symptoms.

## 2. Experimental Section

### 2.1. Preparation of *P. lobata* Total Isoflavones (PTIF)

*P. lobata* was purchased from Kapdang Co. (Seoul, Korea). The sample was identified by Dr. HeeSoon Shin, and a voucher specimen (#NP-1042) was deposited in the Functionality Evaluation Research Group, Korea Food Research Institute, Seongnam, Korea. *P. lobata* (300 g) was extracted with 70% ethanol (3000 mL) for 3 h at 80 °C in a reflux apparatus. The extracts were filtered and concentrated under reduced pressure, and samples were lyophilized to yield a dark yellow powder. The yield of *P. lobata* extracts was 22.8%. The compositional analysis of total isoflavones from *P. lobata* extracts was performed by a high performance liquid chromatography (HPLC) system equipped with a Waters 1525 pump, a 2707 auto sampler and a 2998 PDA detector. The chromatic separation was achieved at 30 °C on Waters Sunfire™ C18 (250 mm × 4 mm i.d., 5 μm particle size) column. The run time was set at 30 min, and the flow rate was 1.0 mL/min. The sample injection volume was 10 μL. The mobile phase was 0.1% (v/v) formic acid (A), 100% acetonitrile (B), filtered through a 0.45 μm filter and degassed prior to use. Separation was achieved with gradient elution using 0.1% formic acid as a solvent. The gradient was reduced by 90% from 0 to 10 min, 75% from 10 to 15 min and 50% from 15 to 20 min and was increased by 90% from 20 to 28 min to equilibrate the column. The flow rate was set at 1.0 mL/min, and samples were detected at 254 nm.

### 2.2. Animals and Treatments

Female Sprague-Dawley (SD) rats, 8-weeks-old, were purchased from Samtako, Gyeonggi-do, Korea. Animals were housed two rats per cage in an air-conditioned room at 23 ± 1 °C, 55%–60% relative humidity and a 12 h light/dark cycle (07:00 lights on, 19:00 lights off) and were given a laboratory regular rodent diet. After acclimatization for 1 week, 9-week-old female SD rats were anesthetized with 2% of isoflurane and the ovaries were removed bilaterally. A sham operation, during which the ovaries were just touched with forceps, was performed on the sham group. One week after surgery, rats were divided into the five following treatment groups: (1) sham + vehicle, (2) OVX + vehicle, (3) OVX + 17β-estradiol (E2, 10 μg/kg once daily, i.p.), (4) OVX + PTIF 30 mg/kg and (5) OVX + PTIF 100 mg/kg. PTIF was dissolved in distilled water for oral administration at the desired doses in a volume of 5 mL/kg once daily. E2 was dissolved in distilled water, with 1% dimethyl sulfoxide (DMSO) and 0.1% Tween 20. All groups were treated for eight weeks. During the experimental period, body weight and femur bone mineral density (BMD) were determined weekly. At the end of the treatment period, the rats fasted for 12 h and blood was collected via the abdominal aorta. Uterus tissue and other organs were dissected, washed with saline solution and weighed for analysis. Uterus and organ indexes (mg/g) were calculated by dividing the uterus and organ weights by the body weight. Liver tissues were collected for the experiments described later in this section. All animal experiments were carried out according to the guidelines of the Korea Food Research Institutional Animal Care and Use Committee.

### 2.3. BMD Measurements

The BMD of femur was measured by a PIXImus (GE Lunar PIXImus, GE Healthcare, WI, USA), dual energy X-ray absorptiometer (DXA), equipped with appropriate software for bone density assessment in small laboratory animals. Calibration of the instrument was conducted as recommended by the manufacturer. Quality control with BMD (0.0553 g/cm^2^) and percentage fat composition (16.7%) of the phantom were also performed each time the instrument was switched on. The percent coefficient of variation (% CV) for rat BMD at the femur was 1.5%–2.0%. All rats were placed in the same direction.

### 2.4. Serum Lipid, Estradiol and Bone Marker Analysis

The serum samples were prepared by centrifugation of the collected blood samples (1013 g for 15 min at 4 °C), then stored at −80 °C for biochemical determinations. The serum concentrations of total cholesterol (TC), triglyceride (TG), high density lipoprotein-cholesterol (HDL-c), alanine transaminase (ALT), aspartate aminotransferase (AST) and alkaline phosphatase (ALP) were determined using an automatic analyzer (ADVIA 1650, Bayer, Tokyo, Japan). Serum LDL-c was estimated using Frieldwann’s equation. Serum hormone level was determined by radioimmunoassay (RIA). The estradiol RIA was performed according to the instructions accompanying a Coat-a-Count kit (Diagnostic Products, Los Angeles, CA, USA). Serum concentrations of the bone formation marker, osteocalcin (OC) [20], were assayed using a rat ELISA kit (Metra OC, Quidel Corporation, San Diego, CA, USA). Serum levels of telopeptides of collagen type I (CTx), which correlate with bone resorption, with high levels indicating excessive osteoclastic activity [21], were analyzed using commercial ELISA kits (Serum CrossLaps, Nordic Bioscience, Herlev, Denmark; Metra Serum Pyd, Quidel Corporation, San Diego, CA, USA). The hepatic lipid was extracted using the procedure developed by Folch *et al.* [22], and TC and TG contents were determined using a commercial enzymatic kit (Asan Pharm. Co., Seoul, Korea). The intra- and inter-assay coefficients of variation (CVs) were 8.35% and 9.45% for estradiol, 6.4% and 8.9% for CTx, 2.8% and 5.2% for OC, 4.5% and 4.8% for TG and 2% and 6% for TC, respectively.

### 2.5. Liver Histological Analysis

The liver tissues were divided into the tissue freezing medium (TBS, Durham, NC, USA) and then deep-frozen in liquid nitrogen for their preservation. The liver tissues were cut into 5 μm-thick sections using a Cryostat instrument (CM 1850; Leica, Heidelberg, Germany). The tissue sections were fixed in 10% neutral phosphate-buffered formalin solution. The lipids and nuclei of the liver cells were stained with hematoxylin and eosin (H & E). A diagnosis of fatty liver was made based on the presence of macro- or micro-vesicular fat in >5% of the hepatocytes in a given slide.

### 2.6. Statistical Analysis

Statistical analysis was performed using GraphPad Prism 5.03 (GraghPad Software, San Diego, CA, USA). The significance of differences among the groups for repeated measurements was analyzed using two-way ANOVA (repeated measurement and time course) followed by Bonferroni posttests or one-way ANOVA followed by Tukey’s test. All data were presented as the mean ± SEM. Values of *p* < 0.05 were considered statistically significant.

## 3. Results

### 3.1. Compositional Analysis of Total Isoflavones from *P. lobata* extracts

**Figure 1:**
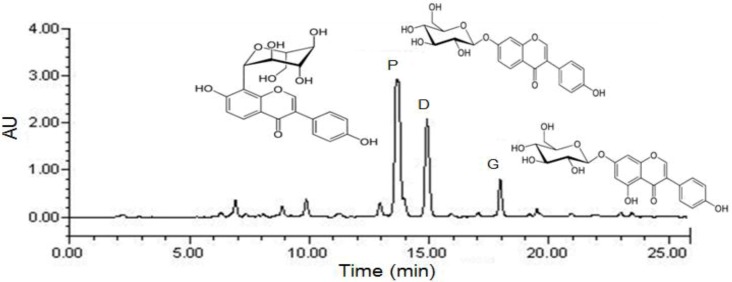
Preparative HPLC chromatography of PTIF. P: puerarin; D: daidzin; G: genistin; PTIF: *P. lobata* total isoflavones.

The HPLC chromatogram of total isoflavones is shown in Figure 1, and the puerarin, daidzin and genistin concentrations in the *P. lobata* extracts are listed in Table 1. After purification, puerarin (7.2%) was the major compound in the extract, which also contained daidzin (3.8%) and genistin (1.5%). The PTIF for oral administration was calculated based on its isoflavone contents.

**Table 1:** Composition of puerarin and other isoflavones from *P. lobata* extracts.

Isoflavones	Concentration (%, w/w)
Puerarin	7.2
Daidzin	3.8
Genistin	1.5
Total isoflavones	12.5

### 3.2. Effects of PTIF on Body Weight and Serum Lipid Concentrations in OVX Rats

**Figure 2:**
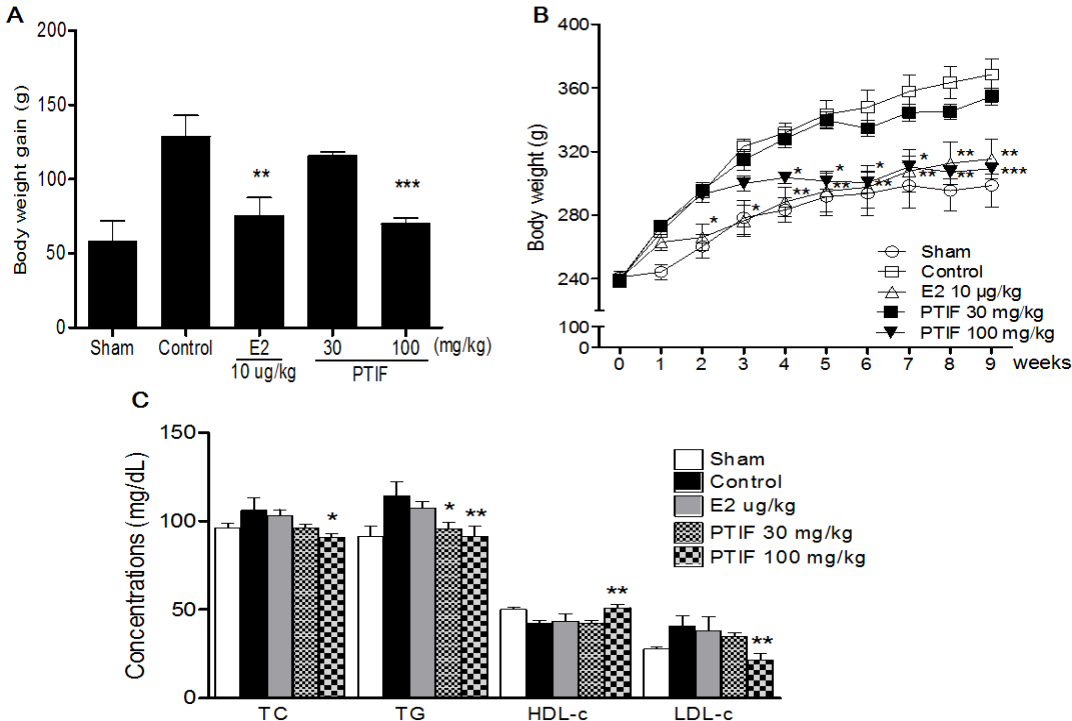
Effects of PTIF on body weight gain (**A**), weekly body weight (**B**) and serum lipid concentrations (**C**) in ovariectomized (OVX) rats. The body weight gain was calculated by the equation: final body weight − initial body weight. All data are the mean ± SEM values (*n* = 12 per group). Body weight was statically significant *vs*. control by repeated-measure ANOVA. Body weight gain and serum concentration were compared with control (one-way ANOVA). * *p* < 0.05, ** *p* < 0.01 and *** *p* < 0.001, significantly different from the OVX-control group.

The body weights increased over time in all groups, but body weights increased significantly more in the OVX-control group alone compared to the sham group. A significant difference in body weight was observed between the E2 10 μg/kg treated group and the OVX-control group by two weeks after initiating administration (Figure 2B). As expected, the body weight of the E2 10 μg/kg treated group was significantly less than that of the OVX-control group. Furthermore, the body weight gain of the PTIF 100 mg/kg treated group was significantly less than that of the OVX-control group. The PTIF 100 mg/kg treated group showed similar results with the E2 treated group (Figure 2A). Serum TG, TC and LDL-c levels were significantly higher in the OVX-control group compared with the Sham group. After eight weeks of treatments, the PTIF 30 and 100 mg/kg treated groups showed significantly lower serum TG, TC and LDL-c levels in a dose-related manner, while causing the reverse on serum HDL-c (Figure 2C).

### 3.3. Effects of PTIF on OVX-Induced Hepatic Steatosis in Rats

**Figure 3:**
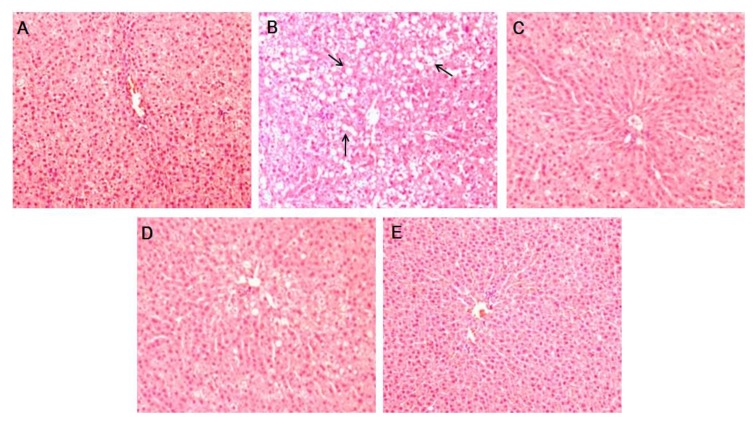
Histological images of liver tissues from OVX-induced hepatic steatosis displaying the hepatoprotective effects of PTIF. There were no histological abnormalities observed in sham rats (**A**). OVX-induced hepatic steatosis showing hepatocytes with severe cytoplasmic vacuoles and swelling (**B**). E2 10 μg/kg resulted in the prevention of hepatic fatty deposition in hepatocytes (**C**). The treatments with PTIF 30 and 100 mg/kg resulted in the prevention of hepatic fatty deposition in hepatocytes (**D**,**E**). The tissues were surgically excised and subjected to histological study by staining with hematoxylin and eosin (H & E). All magnifications: 200×. The arrows indicate fatty hepatocytes.

As shown in Table 2, PTIF exhibits a hepatoprotective effect in OVX-induced hepatic steatosis, indicated by reduced hepatic lipid contents and serum ALT and AST levels. It was found that OVX-control rats alone developed a high degree of steatosis, with severe cytoplasmic vacuoles and hepatocyte swellings (Figure 3B), whereas no histological abnormalities were observed in sham rats (Figure 3A). The treatments of PTIF resulted in the prevention of hepatic fatty deposition in hepatocytes (Figure 3D,E).

**Table 2:** Effects of PTIF on OVX-induced hepatic steatosis. Data are the mean ± SEM values (*n* = 12 per group). ^a^ A significant decrease at *p* < 0.05, when compared with the control group; ^b^ A significant decrease at *p* < 0.01, when compared with the control group; ALT, alanine transaminase; AST, aspartate aminotransferase; TC, total cholesterol; TG, triglyceride.

Groups	Serum (mg/dL)		Liver lipid (mg/g wet wt)	
	ALT	AST	TC	TG
Sham	109.1 ± 12.5	72.1 ± 6.9	7.1 ± 0.7	35.2 ± 4.9
Control	135.5 ± 15.7	94.5 ± 6.6	11.1 ± 0.8	68.8 ± 7.4
E2 10 μg/kg	121.1 ± 17.4 ^a^	80.3 ± 4.9 ^a^	8.2 ± 0.6 ^a^	51.8 ± 6.8 ^a^
PTIF 30 mg/kg	120.6 ± 19.5 ^a^	77.5 ± 6.8 ^a^	7.5 ± 0.5 ^a^	48.1 ± 5.8 ^a^
PTIF 100 mg/kg	118.4 ± 13.5 ^b^	76.5 ± 6.8 ^b^	6.5 ± 0.5 ^b^	43.1 ± 5.8 ^b^

### 3.4. Effects of PTIF on BMD and Organ Weights in OVX Rats

**Figure 4:**
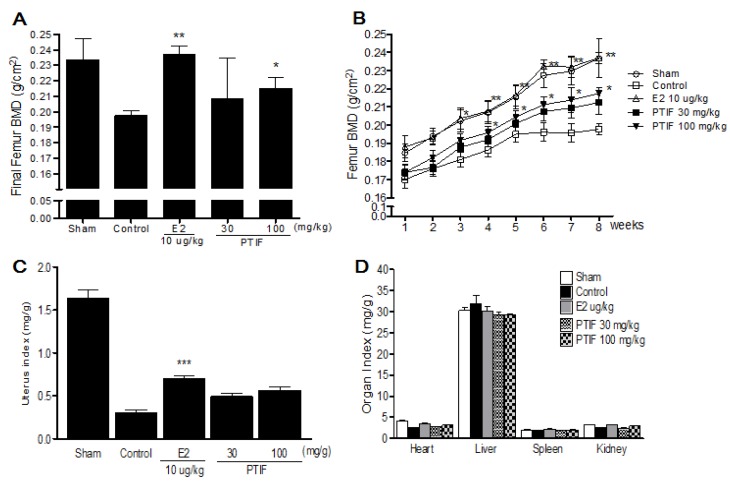
Effects of PTIF on final femur one mineral density (BMD) (**A**), femur BMD changes (**B**), uterus index (**C**) and organ index (**D**). Data are the mean ± SEM values (*n* = 12 per group). Femur BMD changes were statically significant *vs*. control by repeated-measure ANOVA. Final femur BMD and organs weights were compared with control (one-way ANOVA). * *p* < 0.05, ** *p* < 0.01 and *** *p* < 0.001, significantly different from the OVX-control group.

Three weeks after the OVX operation, OVX groups showed a significant decrease in the femur BMD compared to the sham group (Figure 4B). After eight weeks of treatments, the final femur BMD of the PTIF 100 mg/kg treated group was significantly higher than that of the OVX-control group (Figure 4A). OVX caused atrophy of uterine tissue, indicating the success of the surgical procedure and in the E2 10 μg/kg treated group, the uterus index (mg/g) increased significantly compared to the OVX-control group. However, the PTIF 30 and 100 mg/kg treated groups did not show an effect on the uterus index following OVX operation (Figure 4C). Also, the index of heart, liver, spleen and kidney was not significantly different in each group, either (Figure 4D).

### 3.5. Effects of PTIF on Bone Marker in OVX Rats

**Figure 5:**
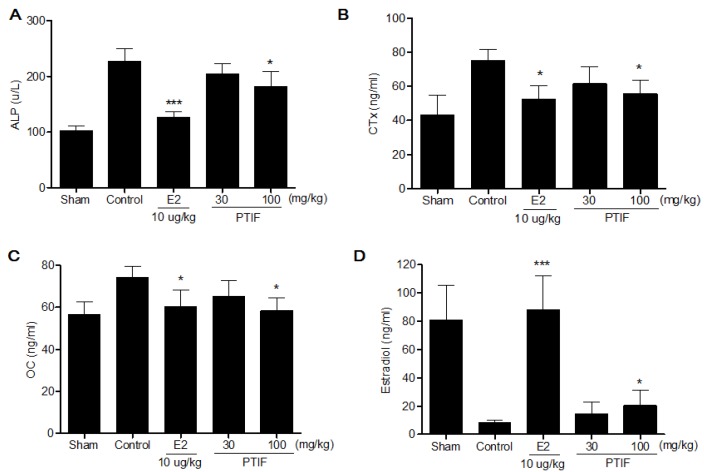
Effects of PTIF on serum alkaline phosphatase (ALP) (**A**), collagen type I (CTx) (**B**), osteocalcin (OC) (**C**) and estradiol (**D**) levels. Data are the mean ± SEM values (*n* = 12 per group). * *p* < 0.05 and *** *p* < 0.001, significantly difference from the OVX-control group.

ALP, CTx and OC levels in the OVX-control group were significantly higher compared to the sham group. After eight weeks of treatments, the PTIF 100 mg/kg treated group displayed significantly lower ALP, CTx and OC levels compared to the OVX-control group (Figure 5). In addition, the PTIF 100 mg/kg treated group showed significantly higher serum estradiol level compared to the OVX-control group (Figure 5D).

## 4. Discussion

Our results demonstrate that eight weeks of treatment with high-dose *P. lobata* total isoflavones (PTIF, 12.5%, w/w) significantly decreased the BMD loss in the femur and inhibited the increase in body weight and lipoprotein levels compared to the OVX-control group. These findings indicate that PTIF induced multiple metabolic changes in a rat model of postmenopausal symptoms.

Bone loss caused by estrogen deficiency in both humans and experimental animals is primarily due to an increase in osteoclastic bone resorption [23]. OVX rats, which exhibit most of the characteristics of human postmenopausal osteoporosis [19] have been widely used as a model for the evaluation of potential osteoporosis treatments [24].

Like previous reports, our OVX resulted in a significant decrease in the femur BMD after eight weeks. This BMD loss was accompanied by a significant increase in bone remodeling, as was evidenced by the enhanced bone turnover makers. An increase in ALP serum levels, the most widely used biochemical bone turnover marker [25], was observed in OVX rats [26,27]. Furthermore, an increase in serum levels of telopeptides of collagen type I (CTx), which correlate with bone-resorption, with high levels indicating excessive osteoclastic activity and bone formation marker osteocalcin (OC) were observed in OVX rats, and these results were supported by Hertrampf *et al.* [28]. Although we did not determine the 3D architecture of trabecular bone within the distal metaphyseal femur region, eight weeks of treatment with high-dose PTIF significantly decreased the BMD loss in the femur, which was reflected by the decrease in ALP, CTx and OC serum levels compared to the OVX-control group; this effect may have been due to decreased bone resorption.

OVX dramatically increases body weights, while E2 treatment maintains normal levels completely [29]. Although the mechanisms by which OVX induces increases in body weight are not clear, estrogen deficiency induced body fat accumulation and, subsequently, caused an increase in body weight [30]. Heine *et al.* [31] demonstrated that estrogen receptor (ER) knockout mice have higher fat mass and lower energy expenditure than wild-type mice. Estrogen may be involved directly in energy metabolism of rats by binding to ER within the abdominal and subcutaneous fat tissues [32]. In our results, eight weeks of treatment with high-dose PTIF inhibited the increase in body weight and serum lipid concentration. These results suggest that high-dose PTIF acts as a source of proestrogenic compounds based on their effects on lipid metabolism in OVX rats.

Estrogen is mainly metabolized into sulfate conjugate, which is catalyzed by sulfotransferase (SULT) [33]. The SULT1E1 enzyme of the estrogen-preferring SULT family 1E is a key plasma estrogen SULT that determines the activity of estrogen [34]. Lee *et al*. [35] reported that SULT1E1 might be related to the induced ability of estrogen activation, potentially affecting bone mineral turnover and increasing the concentration of BMD in postmenopausal women. From the above reports, it is hypothesized that high-dose phytoestrogen supplements could increase active estradiol levels, at least in part, through the SULT1E1 gene expression in OVX rats. Our serum estradiol level results were supported by the previous reporting that administration of herbal extracts, including isoflavones, showed slightly or significantly higher estradiol levels in OVX rats [36,37,38,39].

We found in the study that there was an obvious lipid metabolic disturbance in OVX rats. Eight weeks after the operation, the TC and TG concentration in liver in OVX control group was found obviously higher than those in other groups. Although the mechanisms by which OVX induces TC and TG concentration in liver, the increased insulin concentration in OVX rats may accelerate the dephosphorylation effect of the HMG-CoA reductase, which is a rate-limiting enzyme of the composition of cholesterol in liver [40]. Serum AST and ALT levels are clinically and toxicologically important indicators [41] and increase as a result of tissue damage caused by toxicants or disease conditions. In our results, PTIF caused no apparent liver toxicity, based on lower plasma AST and ALT. Moreover, high-dose PTIF exhibits a hepatoprotective effect, indicated with improved hepatic lipid contents in OVX rats. These results suggest that high-dose PTIF could regulate the lipid metabolic disturbance in liver in OVX rats.

## 5. Conclusions

In conclusion, our results demonstrated that PTIF has multiple metabolic benefits in OVX rats. The treatment of PTIF led to increased serum estrogen levels, decreased serum lipoprotein levels and significantly improved femur BMD, without elevating the serum levels of the liver enzymes, AST and ALT. Taken together, our findings suggest that PTIF may be useful for controlling lipid and bone metabolism, at least in OVX rats. Further research is necessary to determine whether PTIF will have the same effects in humans.
